# Associations of Serum Calprotectin, Arterial Stiffness and Long COVID Symptoms in Dalmatian Kidney Transplant Recipients

**DOI:** 10.3390/v15081776

**Published:** 2023-08-21

**Authors:** Tina Đogaš, Ivana Novak, Marija Babić, Marijana Vučković, Leida Tandara, Josipa Radić

**Affiliations:** 1 Internal Medicine Department, Nephrology and Hemodialysis Division, University Hospital of Split, 21000 Split, Croatia; tina.dogas@gmail.com (T.Đ.); ivana.i.novak@gmail.com (I.N.); marijaterezababic92@gmail.com (M.B.); mavuckovic@kbsplit.hr (M.V.); 2 Department of Medical Laboratory Diagnostics, University Hospital of Split, University of Split School of Medicine, 21000 Split, Croatia; ltandara@kbsplit.hr; 3 Department of Internal Medicine, University of Split School of Medicine, 21000 Split, Croatia

**Keywords:** long COVID pathophysiology, neutrophil activation, serum calprotectin, kidney transplantation, kidney rejection

## Abstract

We aimed to explore long COVID symptoms, serum calprotectin levels, and the parameters of arterial stiffness in Dalmatian kidney transplant recipients (KTRs) and their possible associations. A cross-sectional, single-center case-control study on 98 KTRs who had recovered from COVID-19 was performed. Long COVID symptoms were explored via standardized questionnaires assessing quality of life, and serum calprotectin was also measured. Out of 98 KTRs with a mean age of 62 years, 63 (64.3%) were men. Medical history, clinical and laboratory parameters, and arterial stiffness measurements were obtained for each study participant. Difficulties with mobility were present in 44.3% of the KTRs, while difficulties with self-care were present in 6.2%, difficulties with usual activities were demonstrated by 35.1%, pain in the extremities was present in 52.5%, and anxiety and depression were present in 26.8%. Our results showed significant differences regarding serum calprotectin levels in clinical manifestations of acute COVID-19 and follow-up laboratory parameters. The most significant positive predictors of the serum calprotectin value in the KTRs were respiratory insufficiency, acute kidney failure, the prescription of antihypertensives, leukocyte and neutrophil counts, the neutrophil/lymphocyte ratio and lactate dehydrogenase levels. Negative predictors were the time since COVID-19, high-density lipoprotein levels, kidney function parameters, and the lymphocyte count. To conclude, serum calprotectin has emerged as a possible promising biomarker for subclinical allograft rejection; however, further studies are needed to better understand this subject.

## 1. Introduction

Long COVID syndrome, or post-acute sequelae of the SARS-CoV-2 infection (PASC), has become a meaningful public health concern. The latest consensus on the definition of long COVID syndrome emerged in October 2021 when the World Health Organization (WHO) proposed a definition for what they referred to as the post-COVID-19 condition as the presence of symptoms that manifest within 3 months of acute illness, last for at least 2 months, and cannot be explained by an alternative diagnosis [1]. The reported average prevalence of long COVID syndrome ranges from 3 to 11.5% and has had a substantial deleterious impact on social and professional functioning and day-to-day activities [1]. A systematic review of 57 peer-reviewed studies that included 250,351 survivors of COVID-19 showed that the median age of participants was 54.4 years, where 56% were men and 54% suffered at least one PASC symptom 6 months after the illness [2]. Long COVID is a multisystem disease that develops regardless of the initial disease’s severity [3,4]. The female sex and previous comorbidities emerged as the most prominent risk factors for long COVID syndrome, while age and initial disease severity yielded ambiguous results [5,6,7]. Kidney transplant recipients (KTRs) are at particularly high risk of unfavorable outcomes from COVID-19, showing fatality rates that are at least ten times higher, which is probably due to the chronic use of immunosuppressants and the higher number of comorbidities [8]. There are only a few studies that have explored the prevalence of and risk factors for long COVID syndrome in a population of KTRs, and these studies were mostly conducted in the pre-vaccination and pre-Omicron era [8,9]. In the largest prospective cohort of KTRs, the prevalence of at least one long COVID symptom persisting for 12 weeks was 27% [8]. Neither any baseline characteristics nor the severity of acute COVID-19 were found to be independent predictors of long COVID syndrome except for a higher total burden of symptoms in the acute phase [8]. The clinical spectrum of long COVID syndrome is wide and comprises fatigue, headache, brain fog, sleep disturbances, musculoskeletal pain, shortness of breath, chest pain, and dysautonomia [3,4]. In the cited cohort of KTRs, the most frequent symptoms were body aches, which is followed by weakness and headache [8]. The pathophysiology is still unclear, as it cannot be explained solely by organ damage-related symptoms; therefore, long-lasting inflammatory mechanisms have been proposed [1]. While there are no firm correlations between anti-SARS-CoV-2 humoral responses and the severity of self-reported symptoms, calprotectin autoantibodies have proven to be highly associated with clinical recovery and feeling “normal” post-infection, suggesting an increased autoantigen presence [10]. Previously, calprotectin, as a signature of neutrophil activation, emerged as a promising biomarker for risk stratification in acute COVID-19, accurately predicting disease severity and the need for mechanical ventilation and admission to the intensive care unit (ICU) at an early stage in the emergency department [11]. An infection with SARS-CoV-2 results in the activation of innate and adaptive immune responses, which, when dysregulated, cause local and systemic tissue damage [12]. Tissue damage sustains and promotes the amplified autocrine production of calprotectin, resulting in a hyperinflammatory loop that prevents its protective function [9]. An aberrant immune response results in an increase in the neutrophil count due to the presence of aberrant, immature neutrophil subsets and the depletion of lymphocytes and NK cells [12]. The activation of neutrophils plays a central role in the immunopathology of COVID-19 [11]. Neutrophil extracellular traps (NETs) have been considered drivers of endothelial damage and can cause thrombosis by blocking capillary circulation [13]. The crosstalk between neutrophils, calprotectin, platelets, and endothelial cells appears to play a crucial role in both the arterial and venous thrombotic complications of COVID-19 [14]. Reports on the circulating calprotectin in KTRs are scarce. It was once speculated that the combined use of serum calprotectin and procalcitonin could be a diagnostic marker of allograft rejection, allowing one to distinguish between allograft rejection and non-viral inflammatory processes [15,16]. Another study examining leukocyte–transendothelial-migration-related allograft rejection showed that the use of calprotectin as a surrogate marker preceded conventional clinical models, such as an increase in serum creatinine levels or a reduction in the daily volume of urine, using a median of 5 days, suggesting a high predictive value for allograft rejection since it is apparently not significantly influenced by other common confounders during a delayed graft function (DGF) in the first 4 weeks after kidney transplantation [17]. Calprotectin is a member of the calcium-binding S100 protein family. It is a heterodimer of two subunits, S100A8/S100A9, which are also known as myeloid-related proteins 8 and 14 (MRP8 and MRP14), implying their molecular masses of 8 kD and 14 kD, respectively [17,18]. Calprotectin is abundant in the cytoplasm of neutrophils and monocytes, forming 45% of cytosolic proteins in neutrophils [18]. It is an acute phase reactant with a half-life of 5 h and is produced locally at the inflammatory site [19]. In addition to its intracellular role in calcium-dependent signaling, cell differentiation, cell cycle progression, the regulation of kinase activities, cytoskeleton–membrane interactions, phagocytes trafficking, and arachidonic acid metabolism, the circulating calprotectin acts as a damage-associated molecular pattern (DAMP) or alarmin, interacting with pattern recognition receptors (PRRs) and thus activating downstream inflammatory signal pathways [12,17,18,19]. Calprotectin is an endogenous ligand of Toll-like receptor 4 (TLR-4) and is a receptor for advanced glycation end products (RAGE), promoting NF-κB signaling and pro-inflammatory responses [12,18]. Therefore, calprotectin is a major component in NETs generated to trap and destroy pathogens in the extracellular space [13]. Its calcium and zinc-binding antimicrobial properties limit essential nutrients and, hence, the growth of invading pathogens [13]. Furthermore, this protein has strong apoptosis-inducing activity in cell cultures [13]. Circulating calprotectin increases following infection and trauma as well as in a wide variety of metabolic, inflammatory, and autoimmune conditions, such as inflammatory bowel disease (IBD) and rheumatoid arthritis (RA), where it can be considered a diagnostic marker of disease activity in response to the treatment and monitoring of disease remission or recurrence [18,19]. The aim of this study was to explore long COVID symptoms, including serum calprotectin levels and the parameters of arterial stiffness in Dalmatian KTRs and their possible associations. Serum calprotectin was used as a surrogate for neutrophil activation. To the best of our knowledge, this is the first study to investigate the presence of low-grade neutrophil activation as an underlying mechanism of long-term post-COVID-19 complications in general, not only in KTR survivors.

## 2. Materials and Methods

### 2.1. Study Design and Population

**Figure 1:**
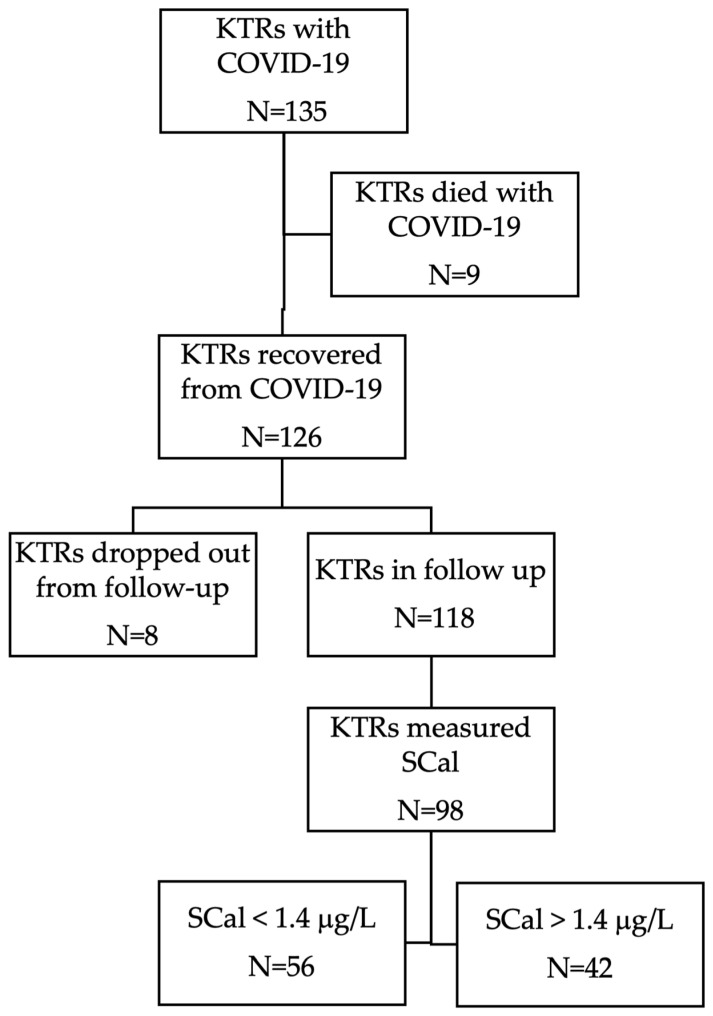
Study flowchart. Abbreviations: KTRs—kidney transplant recipients; SCal—serum calprotectin, N—number.

A cross-sectional study was carried out at the Outpatient Transplant Clinic of the Nephrology Division, Internal Medicine Department, University Hospital of Split, between November 2022 and January 2023. Out of the 135 KTRs with confirmed COVID-19, 98 KTRs recovered from COVID-19 were sampled for serum calprotectin in the follow-up and were included in this study as shown in Figure 1.

KTR COVID-19 survivors regardless of their need for initial hospitalization who were willing to participate and remained in the follow-up were included in this study.

Exclusion criteria were ongoing allograft rejection, malignancy, and symptomatic infection.

All participants provided written consent after being informed about the nature and purpose of the study.

The study protocol was approved by the Institutional Ethics Committee and was performed following the guidelines of the latest version of the Declaration of Helsinki.

### 2.2. Medical History, Clinical and Laboratory Parameters, and Arterial Stiffness Measurements

Data on the participant’s demographics, comorbidities, medications and clinical features, complications, and treatment interventions in acute COVID-19 infection were collected from electronic medical records and were combined with the participant interview and clinical examination conducted by a nephrologist at the outpatient follow-up visit.

Basal triple immunosuppression comprising a calcineurin inhibitor or, less often, an mTOR inhibitor as the mainstay and mycophenolate–mofetil or mTOR inhibitor as the adjacent with an add-on of minimal steroid doses of prednisone at 5 mg or equivalent were adjusted in the acute phase according to COVID-19 severity. The mainstay immunosuppressant was generally continued, targeting trough levels of the therapeutic range, while adjacent immunosuppressant was withheld, halved, or minimized along with the escalation of steroid doses.

After the resolution of acute COVID-19, basal triple immunosuppressant therapy was resumed.

Screening for long COVID was conducted using standardized lifestyle questionnaires of the 5-level EuroQual group-5D (EQ-5D-5L), which comprises 5 dimensions including mobility, self-care, usual activities, pain/discomfort, and anxiety/depression using the EQ visual analogue scale (EQ VAS) to record participant’s self-rated health on a vertical visual analogue scale where the endpoints were between the best and the worst health imaginable. This could be used as a quantitative measure of health outcome reflecting the patient’s own judgment, and the Modified Medical Research Council Dyspnea Scale (mMRC) assessed the degree of baseline functional disability due to dyspnea, which was validated in patients with respiratory diseases as chronic obstructive pulmonary disease (COPD).

The Charlson Comorbidity Index (CCI), which predicts 10-year survival in patients with multiple comorbidities was calculated for each participant. The score consists of 17 variables, including age, history of myocardial infarction, chronic heart failure, peripheral vascular disease, cerebrovascular accident or transient ischemic attack, hemiplegia, chronic cognitive deficit, COPD, connective tissue disease, peptic ulcer disease, liver disease, chronic kidney disease, diabetes, solid and hematological neoplasms, and AIDS. Each variable is graded, and a higher total score indicates a higher mortality rate. The estimated 10-year survival could be calculated using the following formula, 0.983 ^(eCCI×0.9)^.

Peripheral and central blood pressure and arterial stiffness measurements were performed using the Agedio B900 (IEM, Stolberg, Germany) device based on the principle of oscillometry. The right-sized cuff was selected according to the upper arm circumference. All measurements were conducted in a relaxing environment, participants were comfortably seated with their back and arm supported, with feet flat on the ground, legs not crossed, and on an empty bladder. Data on peripheral and central systolic and diastolic blood pressure and pulse wave velocity (PWV; m/s) were obtained.

Blood samples for complete blood count, coagulation profile, and serum biochemical analyses were collected in the Medical Laboratory Diagnostic Division at the University Hospital of Split, Croatia. Serum samples for calprotectin measurement were stored at −20 °C and analyzed using a particle-enhanced turbidimetric immunoassay (BÜHLMANN Diagnostic Corp) on the Roche Cobas 6000, c501 module. The results were expressed as the median (IQR) in μg/L. The high-sensitivity C-reactive protein (hsCRP) was determined by immunoturbidimetry (Roche Cobas 6000; c501 module). The estimated glomerular filtration rate (eGFR) was calculated using the Chronic Kidney Disease Epidemiology Collaboration (CKD-EPI) equation (mL/min/1.73 m^2^). Serum creatinine was measured using the compensated Jaffe method.

Acute kidney injury (AKI) was defined according to KDIGO guidelines as an increase in serum creatinine by ≥26.5 μmol/L or ≥1.5 times the baseline. Allograft outcomes were defined as recovery, injury, and failure.

### 2.3. Statistical Analysis

Categorical data were presented by absolute and relative frequencies. The normality of the distribution of continuous variables was tested by the Shapiro–Wilk test. Continuous data were described by the median and the limits of the interquartile range (IQR). The Mann–Whitney U-test was used to compare the median between the two groups, and the Kruskal–Wallis test (post hoc Conover) was used in the extension. Fisher’s exact test was used to analyze the differences between proportions. The correlation between numeric variables was evaluated by Spearman’s correlation coefficient ρ (rho). The serum calprotectin variable was categorized into two groups with a cut-off value of 1.4 μg/mL. Logistics regression analysis (bivariate and multivariate–stepwise method) was used to analyze the independent factors associated with higher concentrations of serum calprotectin. The results of logistic regressions were presented with odds ratios (ORs) and 95% confidence intervals (CIs). All *p*-values were two-sided. The level of significance was set at an α of 0.05. The statistical analysis was performed using the MedCalc^®^ Statistical version 19.6 (MedCalc Software Ltd., Ostend, Belgium; https://www.medcalc.org; 2020) and IBM SPSS Stat. 23 (IBM Corp. Released 2015. IBM SPSS Statistics for Windows, Version 23.0. Armonk, NY, USA) assessed on 4 April 2023.

## 3. Results

### 3.1. Long COVID Syndrome

Mild and moderate mobility difficulties were experienced by 16 (16.5%) KTRs each and severe difficulties were experienced by at least 11 (11.3%) KTRs. Mild, moderate, and severe self-care difficulties were reported for three (3.1%), two (2.1%), and one (1%) KTRs, respectively. Mild difficulties with daily activities were reported for 12 (12.4%), moderate 16 (16.5%), severe 5 (6.2%) and extreme only 1 (1%) KTRs. Mild, moderate, severe, and extreme pain in their extremities were found to be struggling for 15 (15.5%), 22 (22.7%), 10 (10.3%), and 4 (4.1%) KTRs, respectively. Mild, moderate, and severe anxiety and depression were reported for 18 (18.6%), 7 (7.2%), and 1 (1%) KTRs.

Serum calprotectin was measured in 98 KTRs who had recovered from COVID-19. In total, 63 (64.3%) KTRs were men. The median serum calprotectin was 1.7 μg/L, the interquartile ranged from 1.1 to 3.45 μg/L, and the minimum maximum ranges were from 0.2 to 14.4 μg/L.

KTRs were divided into two groups according to the serum calprotectin cut-off value that was set at 1.4 μg/L due to the sample distribution characteristics.

The participants’ demographics and basic characteristics are shown in Table 1.

There were no statistically significant differences between the groups of KTRs with higher and lower serum calprotectin levels regarding underlying kidney diseases and associated comorbidities expressed by the CCI, as well as the allograft outcomes.

Underlying kidney diseases were defined as glomerulonephritis (22.4%), vasculitis (1%), diabetes (5.1%), nephroangioslerosis (6.1%), polycystic kidney diseases (16.3%), reflux nephropathy (9%), other (6%) and unknown (33.7%) kidney conditions.

Of the associated comorbidities, hypertension was experienced by 90 (91.8%) KTRs, while diabetes was experienced by 27 (27.6%), cardiovascular diseases were experienced by 26 (26.5%), heart failure was experienced by 23 (23.5%), chronic obstructive pulmonary disease was experienced by 7 (7.1%), hyperlipidemia was experienced by 67 (68.4%) solid malignancies were experienced by 8 (8.2%), skin malignancies were experienced by 16 (16.3%), and the monoclonal gammopathy of undetermined significance was experienced by 3 (3.1%) KTRs.

Additionally, there were no statistically significant differences between the groups of KTRs with higher and lower serum calprotectin levels according to long COVID symptoms, as shown in Supplementary Table S1.

The same was observed for the time elapsed from COVID-19 and long COVID symptoms. A trend toward more severe pain in its extremities, implying peripheral neuropathy, was observed in the first 6 months following COVID-19 that was not overall statistically significant (*p* = 0.05).

The highest levels of serum calprotectin were observed in the first 6 months and the lowest was between 6 and 12 months following COVID-19. Surprisingly, a statistically significant so-called second peak or the re-increasing of serum calprotectin levels was observed again beyond 12 months following COVID-19.

Accordingly, a similar trend was observed for central (*p* = 0.02) and peripheral pulse pressure (*p* = 0.008), arterial stiffness parameters (*p* = 0.04), and the CCI (*p* = 0.03), although without reaching statistical significance after 12 months following COVID-19, as shown in Table 2.

No statistically significant differences in peripheral and central blood pressure and arterial stiffness parameters were found between the two groups of KTRs concerning serum calprotectin levels.

A significant proportion of KTRs in our sample was vaccinated against SARS-CoV-2 either before or after COVID-19. In total, 40 (41.2%) KTRs were SARS-CoV-2 naive. Precisely, 57 (58.8%) KTRs had been vaccinated before infection, 6 (6.2%) receiving one, 26 (26.8%) receiving two, and 23 (23.7%) receiving three mRNA vaccines, while only 2 (2.1%) KTRs received two vectors’ vaccines. After infection, one mRNA vaccine was received by three participants (3.1%), two were received by seven participants (7.2%), three were received by five participants (5.2%), and four were received by one (1%) participant. Vaccination status did not correlate to serum calprotectin levels.

### 3.2. Acute Illness Characteristics

Of the clinical symptoms of acute COVID-19, only shortness of breath was statistically and significantly more frequent in the group of participants with higher serum calprotectin levels (*p* = 0.005). Other symptoms, such as cough, fever, algic syndrome, anosmia, fatigue, diarrhea, and cognitive disturbances, did not show statistically significant differences between the two groups.

There were no statistically significant differences between the two groups of KTRs regarding the severity of initial viral pneumonia, although a binary logistics regression pointed out that patients with bilateral pneumonia had a six times greater chance of higher calprotectin levels 6 to 12 months following COVID-19.

In numbers, 53 (55.2%) KTRs had no pneumonia, among which 34 (60.7%) were in the lower serum calprotectin levels group and 19 (47.2%) KTRs were in the higher serum calprotectin levels group; 16 (16.7%) KTRs had mild pneumonia, among which 11 (19.6%) were in the lower serum calprotectin levels group and 5 (12.5%) KTRs were in the higher serum calprotectin levels group, while bilateral pneumonia developed in 27 (28.1%) KTRs, among which 11 (19.6%) were in the lower serum calprotectin levels group and 16 (40%) KTRs were in the higher serum calprotectin levels group.

On the contrary, statistically significant differences were noticed regarding the development of respiratory insufficiency (*p* = 0.02), and the need for respiratory support (*p* = 0.03) in favor of the group of KTRs with higher serum calprotectin levels.

Respiratory insufficiency developed in eight (8.2%) KTRs, among which seven (16.7%) KTRs had higher serum calprotectin levels and one (1.8%) KTR had lower serum calprotectin levels.

For the majority, 90 (91.8%) KTRs in the sample did not require oxygen supplementation, which is a trend observed with newer virus strains.

Statistically significant and a higher incidence of acute kidney injury was present in the group of KTRs with higher serum calprotectin levels (*p* = 0.03) compared to the group with lower calprotectin levels. The same was not reached for renal replacement therapy (*p* = 0.17).

Acute kidney injury developed in 21 (22.1%) KTRs, among which 13 (33.3%) KTRs had higher serum calprotectin levels and 8 (14.3%) KTRs had lower serum calprotectin levels.

Renal replacement therapy required only two (5%) KTRs in the higher calprotectin levels group. These participants remained dialysis-dependent.

No associations between thromboembolic complications of acute illness and serum calprotectin levels after recovery were found.

Calcineurin inhibitors, the mainstay of immunosuppression, were continued in all 83 (84.7%) participants, with tacrolimus in 58 (60.4%) and ciclosporin in 25 (25.8%), targeting trough levels of the therapeutic range. The same principle was followed in 14 (14.6%) participants with the mTOR inhibitor as their mainstay immunosuppressant. The adjacent immunosuppressant, mycophenolate–mofetil or mTOR inhibitor, was either halved in 18 (19.1%), minimized in 28 (29.8%), withheld in 20 (21.3%) or left unchanged in 27 (28.7%) participants depending on the COVID-19 severity. The unchanged dose of steroids, most often prednisone at 5 mg or equivalent, was continued in 25 (26.3%) participants. Steroid escalations up to 10, 15, 20, and 32 mg were prescribed for 13 (14%), 8 (8%), 19 (20%) and 7 (7.4%) participants, respectively. Escalations of so-called mini steroid boluses were prescribed overall for 19 (22.1%) KTRs, between 32 and 125 mg for 13 KTRs, 125 mg for 6 KTRs, and 250 mg for only 1 KTR. One participant was not prescribed a steroid at all, and for another one, information was missing. In our cohort, azithromycin was prescribed to 51 (59.3%) participants, another or more than one antibiotic was prescribed to 45 (52.3%) participants, remdesivir was prescribed to 35 (37.2%) participants, monoclonal antibodies were prescribed to 5 (7.4%) participants, and intravenous immunoglobulins were prescribed to 6 (6.4%) participants.

None of the therapeutic interventions nor immunosuppressant adjustments in acute illness showed statistically significant differences between the groups of KTRs regarding serum calprotectin levels.

No KTRs were switched with basal immunosuppression following COVID-19. The calcineurin inhibitor was continued in 83 (84.7%) KTRs, tacrolimus in 58 (60.4%), and ciclosporin in 25 (25.8%) KTRs and the mTOR inhibitor was continued in 19 (19.4%) KTRs, everolimus in 16 (16.3%) and sirolimus in 3 (3.1%) KTRs; mycophenolate–mofetil was continued in 86 (87.8%) KTRs and azathioprine was continued in 2 (2%) KTRs with prednisone at 5 mg or equivalent in 90 (91.8%), 10 mg in 5 (5.1%) and none in 3 (3.1%) KTRs, respectively.

### 3.3. Follow-up Laboratory Data

In the group of KTRs with higher serum calprotectin levels, significantly higher leukocyte (*p* = 0.02) and neutrophil counts (*p* = 0.004), CRP (*p* = 0.02), and hsCRP (*p* = 0.005), lactate–dehydrogenase (*p* = 0.009), nitrogenous parameters of renal function and phosphorus (*p* = 0.003) were found, as shown in Table 3 and Supplementary Table S2. On the other hand, lymphocyte count (*p* = 0.005), high-density lipoprotein (*p* = 0.01), and creatinine clearance (*p* = 0.04) values were significantly lower in the group with a higher serum calprotectin level.

Significant correlations of serum calprotectin with laboratory data in Dalmatian KTRs are presented in Table 4.

Accordingly, a positive correlation in serum calprotectin levels was observed for the neutrophil-to-lymphocyte ratio (*p* = 0.004) and CRP-to-albumin ratio (*p* = 0.03).

Both spot urine characteristics and urine cultures did not appear to be correlated to serum calprotectin levels.

The statistically significant associations of serum calprotectin are shown in Table 5.

In our sample population, the most intriguing was a positive correlation with proteinuria (*p* = 0.01) and an inverse correlation with kidney function estimations and measurements (*p* < 0.001).

A bivariate and multivariate stepwise logistics regression were used to predict the probability of higher serum calprotectin levels, as shown in Table 5 and Table 6.

KTRs with a higher leukocyte count had a 1.62 times greater chance of having higher serum calprotectin levels. Inversely, lower eGFR increased the probability of higher calprotectin levels. This model is entirely significant and explains up to 35% (according to Negelkerke R^2^) of the variance of higher calprotectin levels and correctly explains 74% of cases.

A significant positive association of serum calprotectin levels was observed for different antihypertensive drugs, including calcium-channel blockers (*p* = 0.03) moxonidine, (*p* = 0.007) and urapidil (*p* = 0.03), while a marginal association was found for valganciclovir (*p* = 0.05) which was used for cytomegalovirus prophylaxis or treatment. No similar associations were observed for other maintenance immunosuppressants and chronic medications.

## 4. Discussion

To our knowledge, this is the first study to examine serum calprotectin levels as a surrogate for smoldering low-grade neutrophil activation in general and not just in KTRs recovered from COVID-19. The leading hypothesis proposed for the immunobiology of long COVID is that persistent virus or viral antigens and RNAs in tissues drive chronic inflammation, the reactivation of latent viruses, the viral superantigen activation of the immune system or triggering of autoimmunity following acute viral infection, the dysbiosis of the microbiome or virome and unrepaired tissue damage [2,20]. In our survey on long COVID symptoms, we did not establish any significant association between the serum calprotectin levels and the time elapsed from COVID-19. A trend toward more pronounced aching in the extremities suggests that an increased prevalence of peripheral neuropathy in the first 6 months following COVID-19 was not overall statistically significant. Neuromuscular involvement has been documented even among asymptomatic convalescents [21]. None of the prominent risk factors for long COVID syndrome, such as the female sex, age, and prior comorbidities, except for the most severe clinical phenotypes of COVID-19 requiring intensive care, showed any significant differences according to serum calprotectin levels. Such findings could be a result of either the preventive or curative effect of the vaccination against SARS-CoV-2, which has been unanimously reported by the literature [2,22,23,24]. Vaccines can promote the clearance of residual viral antigens and particles, eliminating the cause of chronic inflammation, or vaccine-induced cytokines can reprogram autoreactive and pathogenic lymphocytes, thus shutting down this vicious circle [2]. Studies evaluating the influence of SARS-CoV-2 variants suggest that patients infected with the Omicron variant have lower odds of developing long COVID syndrome with a prevalence of less than 25% [25,26]. Emerging evidence suggests that latent herpesvirus reactivation contributes to long COVID syndrome [20,21]. The reactivation of the latent Epstein–Barr virus (EBV), but not the acute mononucleosis infection, has been reported in long COVID syndrome [27,28,29,30]. Not only EBV reactivation but the immune response to EBV reactivation has been shown to reflect myalgia encephalomyelitis (ME) or chronic fatigue syndrome (CFS)-like symptoms in long COVID syndrome [29,31]. Varicella–zoster virus (VZV) reactivations have also been well-documented following COVID-19 [29]. Athletes and military personnel who are free from chronic illnesses and typically develop only mild acute symptomatology that rarely requires hospital care have a similar prevalence of prolonged symptoms to the general population [3]. A neurotropism of SARS-CoV-2 has been suggested to trigger sustained neuroinflammation through either activation of the microglia or autoimmune reactions [1]. Studies that have assessed the brain metabolism identified the hypometabolic activity of the brain in patients with brain fog and persistent fatigue, while muscle mitochondrial dysfunction-disrupting cellular bioenergetics could contribute to the physical dimension [1]. Interestingly, in our study, serum calprotectin levels followed the bimodal U-curve distribution following COVID-19 with the highest values recorded in the first 6 months and the second peak re-occurring after a year. Reports from the literature evaluating markers of endothelial dysfunction suggested that endotheliitis and chronic coagulopathy gradually improved at the 6-month follow-up but remained impaired compared to healthy controls [32]. The same pattern followed aortic stiffening, which proved to be at least partially reversible in the longitudinal follow-up 48 weeks post-acute illness [33]. The bimodal trend, without reaching statistical significance after 12 months following COVID-19, was observed for the parameters of central and peripheral blood pressure and arterial stiffness. An increased sympathy parasympathetic activity is associated with fatigue in various autoimmune disorders [3]. Dysautonomia appears to be a feature of long COVID syndrome as well. A positive feedback loop exists between the autonomic nervous system and the renin–angiotensin system (RAS) [34]. The downregulation of ACE2 receptors upon SARS-CoV-2 infection leads to the RAS imbalance by triggering pro-inflammatory pathways with sympathetic hyperactivity effects [34]. RAS activation contributes to the pathophysiology of autonomic dysfunction, therefore increasing the risk of cardiovascular diseases and metabolic disorders [34]. As a result, malignant ventricular–arterial coupling may precede heart failure with preserved ejection fraction [32]. A typical post-COVID presentation is postural orthostatic tachycardia syndrome (POTS), which is characterized by an increase in heart rate > 30 bpm on standing without a drop in blood pressure and is accompanied by the shortness of breath, palpitations, and dizziness [3]. Dysautonomia following COVID-19 can be presented through labile blood pressure comprising episodes of hypertension and orthostatic hypotension, with a so-called exaggerated blood pressure response to exercise, where heart rate variability may be used as an indicator [34]. Increasing evidence has suggested that vagal dysregulation follows COVID-19 [3]. When comparing serum calprotectin levels as a surrogate for neutrophil activation in KTRs recovered from COVID-19, no solid correlation was found for the severity of initial viral pneumonia, although sub-analyses have pointed out that patients with bilateral pulmonary infiltrates had a six times greater chance for higher serum calprotectin levels between 6 and 12 months following acute illness. On the contrary, a positive correlation was established for the most severe clinical phenotypes of acute illness, with respiratory insufficiency and the need for respiratory support as well as for acute kidney injury, although not for renal replacement therapy, the latter possibly due to the small sample size of only survivors. Surprisingly, in our study, thrombotic complications did not significantly correlate to serum calprotectin levels after recovery. None of the therapeutic interventions in acute illness, comprising immunosuppressant adjustments, showed any significant correlation with serum calprotectin levels afterward. A more liberal approach to the escalation of steroid doses in acute illness was advocated in our center early in the pandemic when more pathogenic virus strains were predominant, leading to the more frequent occurrence of new-onset diabetes or the worsening of glycemia. In addition to iatrogenic, alternative underlying mechanisms have been proposed as insulin secretory deficits from impaired pancreatic β cells due to either direct viral damage through the expression of angiotensin-converting enzyme-2 (ACE2) receptors or the indirect effects of inflammatory state-like triggering stress hormones with insulin resistance and molecular mimicry [4,35,36]. The immunopathology of COVID-19 consists of two phases. The first viral phase is characterized by viral replication and direct virus-mediated endothelial and tissue damage, which is followed by the second phase of systemic endothelial dysregulation and the recruitment of effector immune cells, causing a local and systemic inflammatory response that can lead to COVID-19-associated immunothrombosis, which persists even after viral clearance [1,37]. The inflammatory and coagulation cascade from complex networks opposes the invasion of pathogens and tissue injury [37]. Emergency myelopoiesis, as an excess of circulating immature neutrophils, monocytes, and myeloid progenitors and imbalanced adaptive immunity with profound T-cell lymphopenia are almost pathognomonic of severe disease [2]. NETs are an important interface between inflammation and thrombosis [37]. The tissue factor is the pivotal trigger of the coagulation cascade and fuel for an escalating feedback loop between inflammation and coagulation [37]. Aggregated NETs associated with endothelial disruption cause neutrophil microvascular embolisms [37]. This is consistent with our previous work, which also emphasized the predictive value of D-dimers for clinical complications of COVID-19 in rehospitalization and long COVID syndrome [38]. As expected, serum calprotectin levels positively correlated with inflammatory parameters but inversely with renal function. The most interesting finding was a strong positive correlation of serum calprotectin levels with KTRs’ basal proteinuria and albuminuria. Since it has been proven not to be significantly influenced by spot urine characteristics or microbiology, serum calprotectin may be a more sensitive indicator of ongoing subclinical rejection compared to standard clinical methods. A positive correlation for different antihypertensive drugs suggesting more prevalent and resistant hypertension in the higher calprotectin levels group could be a marker of subclinical allograft injury as well rather than providing implications of long COVID syndrome. The observed second peak of serum calprotectin levels beyond a year following COVID-19 supports the above hypothesis, considering prior immunosuppressant adjustments and viral immunomodulation. Only a few papers have investigated serum calprotectin as a promising biomarker of allograft rejection [15,16,17]. Acute kidney injury still lacks a prompt and reliable surrogate that could be timely when navigating organ support and rescue treatment. The accurate and timely diagnosis of kidney rejection would enable the optimal guidance of immunosuppressant adjustments, improving overall kidney outcomes. A marginal positive correlation of the serum calprotectin level was observed for valgancyclovir which was used for cytomegalovirus prophylaxis and treatment in the follow-up.

This study has some limitations primarily arising from the cross-sectional design, which prevented us from drawing causal relations. Major limitations also included the small sample size, the single–center nature of the study, and the inability to assemble a control group of KTRs who had not been in contact with SARS-CoV-2. Although we collected information about the times of COVID-19 infection and reinfection, we still lacked strain refinements by genomic sequencing. Information about dates and the types of vaccination against SARS-CoV-2 were collected as well, but a small sample size prevented us from estimating the impact of such a confounder on long COVID symptoms. Other limitations of our study include the lack of data on viremia and antibody titers against herpesviruses and information about donor-specific antibodies. Furthermore, we were unable to assess the causal crosstalk between innate and adaptive immunity since the research protocol did not foresee the measurements of hyperinflammatory cytokines, primarily interleukin 6, which is known to positively correlate with the neutrophil count and the neutrophil to lymphocyte ratio [18,39,40,41,42,43,44]. Still, in our opinion, insights into the pathophysiology of long COVID syndrome are valuable with the emergence of re-questioning serum calprotectin as a promising biomarker for subclinical allograft rejection.

## 5. Conclusions

Long COVID symptoms cannot be attributed to smoldering low-grade neutrophil activation with serum calprotectin as a surrogate biomarker. Surprisingly, in our study, serum calprotectin emerged as a possible promising biomarker for subclinical allograft rejection, which is consistent with only a few previous reports. Additionally, long COVID follow-up standardized questionnaires and protocols are needed to ensure more homogenous data across studies.

## Figures and Tables

**Table 1:** Differences in participants’ demographic data and basic characteristics regarding the level of serum calprotectin.

	Median (IQR)			p *
	Low Calprotectin ≤1.4 (n = 56)	High Calprotectin >1.4 (n = 42)	Total (n = 98)	
Height (cm)	174 (166.25–179)	178 (168.75–184.5)	176 (168–183)	0.10
Weight (kg)	76 (70–87.75)	83.5 (70–92)	80 (70–90)	0.18
BMI (kg/m^2^)	25.25 (23.1–28.4)	26.19 (23.68–28.7)	25.55 (23.48–28.4)	0.7
Age (years)	63.5 (53–69.75)	60 (51.75–67.25)	62 (53–69)	0.32
Time since TX (years)	8 (4–12)	6 (3.6–12)	7 (4–12)	0.46
Dialysis duration (years)	2.5 (1.5–5)	2 (0.96–4.75)	2.13 (1.15–5)	0.17
Basal proteinuria (mg/L)	165.5 (78–347.25)	248 (154.5–557)	218 (92–376)	**0.009**
Basal albuminuria (mg/L)	21 (8–138)	61 (19.5–384.5)	34.5 (11.25–163.75)	**0.01**
Basal eGFR CKD-EPI (mL/min/1.73 m^2^)	54.33 (44.1–72.79)	46.17 (28.9–60)	50.9 (38.63–64)	0.25

* Mann–Whitney U-test. Abbreviations: IQR; interquartile range; cm, centimeters; kg, kilograms, BMI; body mass index; eGFR CKD-EPI, estimated glomerular filtration rate chronic kidney disease epidemiology collaboration equation, TX; kidney transplantation.

**Table 2:** Differences in the parameters of central and peripheral blood pressure and arterial stiffness regarding the time elapsed from acute COVID-19.

	Median (IQR)				*p* *
	<6 Months	6–12 Months	>12 Months	Total	
Peripheral SBP (mmHg)	144 (133–155.5)	136 (128–150)	139 (131.5–154.2)	139 (130–151)	0.23
Peripheral DBP (mmHg)	88 (78.5–98)	91 (83–100)	88 (82–98.2)	88 (81–99)	0.64
Peripheral MAP (mmHg)	114 (105.5–123.5)	112 (104–123)	111.5 (107.7–121.7)	112 (104–123)	0.89
Peripheral PP (mmHg)	57 (50–66.7)	46 (37–54)	52 (42.75–62)	51.5 (42.75–59.5)	**0.008** **^†^**
Central SBP (mmHg)	148.5 (135.75–161.7)	139.5 (123.7–152.2)	142 (128–151)	141.5 (128.5–155.25)	0.21
Central DBP (mmHg)	89.5 (80.75–99.2)	91 (84–101.25)	89 (84–102)	90 (82.25–100)	0.68
Central PP (mmHg)	61.5 (45.5–70.5)	48 (36–56.5)	51 (43–60)	51 (39–62.75)	**0.02** **^†^**
HR/min	72 (61.5–82)	74 (67–83)	76 (69–86.25)	74 (66–83)	0.39
Augmentation pressure (mmHg)	16 (8–24)	10 (5–15)	13 (7–19)	12 (7–18.75)	**0.04** **^†^**
Augmentation index (%)	25 (20.5–32)	23 (12–29)	30 (20.75–35)	24 (17–32)	0.20
Reflection coefficient (%)	67 (57–70)	62 (51–71)	63.5 (57.5–69)	63 (56–70)	0.42
PWV (m/s)	10.3 (8.45–11.15)	8.6 (6.9–10.1)	9.45 (8.08–10.2)	9.3 (7.7–10.5)	**0.04** **^†^**
Charlson Comorbidity Index	6 (5–7.25)	4 (3–6)	5 (3.75–7)	5 (3–7)	**0.03** **^†^**

* The Kruskal–Wallis test (post hoc Conover). **^†^** at the significance level of *p* < 0.05 where significantly higher values were found in the group of participants within <6 months following acute COVID-19 than in the group of participants 6–12 months following COVID-19. Abbreviations: IQR, interquartile range; SBP, systolic blood pressure; DBP, diastolic blood pressure; PP, pulse pressure; MAP, mean arterial pressure; HR, heart rate; min, minute; mmHg, millimeters of mercury; PWV, pulse wave velocity.

**Table 3:** Differences in laboratory data according to serum calprotectin level.

	Median (IQR)			*p* *
	Low Calprotectin ≤1.4 (n = 56)	High Calprotectin >1.4 (n = 42)	Total (n = 98)	
Leukocytes (×10^9^)	6.7 (5.73–7.5)	8 (5.78–10.5)	6.95 (5.78–8.73)	0.02
Neutrophils (%)	56.6 (49.7–65.9)	64.05 (57.05–69.68)	60.7 (53.75–67.25)	0.004
Lymphocytes (%)	30.2 (21.9–36)	24.6 (17.3–31.03)	27.9 (20.75–34.05)	0.005
Urea (mmol/L)	7.55 (5.93–10.8)	10.6 (8.08–17.48)	9.1 (6.43–12.35)	0.003
Creatinine (µmol/L)	118.5 (94.5–147)	144 (112.75–210.75)	123.5 (100–169)	0.003
eGFR CKD-EPI (mL/min/1.73 m^2^)	51.6 (39.63–66.15)	41.75 (25.33–56.65)	44.3 (34.58–63.6)	0.007
LDH (U/L)	187.5 (170.75–213.75)	210 (185.5–250.5)	198 (174–229)	0.009
HDL (mmol/L)	1.55 (1.3–1.8)	1.3 (1–1.65)	1.5 (1.2–1.7)	0.01
Phosphorus (mmol/L)	0.99 (0.82–1.15)	1.11 (0.91–1.32)	1.04 (0.87–1.21)	0.04
Calprotectin (µg/L)	1.2 (0.8–1.5)	3.85 (2.58–5.43)	1.7 (1.1–3.45)	<0.001
CRP (mg/L)	1.7 (1–4.85)	3.1 (1.7–8.5)	2.5 (1.2–5.95)	0.02
hsCRP (mg/L)	1.65 (0.73–4.53)	3.43 (1.58–8.9)	1.98 (0.93–5.79)	0.005
Creatinine clearance (mL/min)	58.4 (50.6–78.35)	49.07 (25.22–60.41)	57.63 (45–72.73)	0.04

* Mann–Whitney U-test. Abbreviations: eGFR CKD-EPI, estimated glomerular filtration rate chronic kidney disease epidemiology collaboration equation; LDH, lactate dehydrogenase; HDL, high-density lipoprotein; CRP, C-reactive protein; hsCRP, high-sensitivity C-reactive protein.

**Table 4:** Significant correlations of serum calprotectin with laboratory data.

	Spearman’s Correlation Coefficient Rho
	Calprotectin
Basal proteinuria (mg/L)	0.181 (0.17)
Basal albuminuria (mg/L)	0.245 (0.07)
Leukocytes (×10^9^)	0.279 (0.01)
Neutrophils (%)	0.384 (<0.001)
Lymphocytes (%)	−0.373 (<0.001)
Glucose (mmol/L)	0.222 (0.03)
Urea (mmol/L)	0.378 (<0.001)
Creatinine (µmol/L)	0.321 (<0.001)
eGFR CKD-EPI (mL/min/1.73 m^2^)	−0.295 (<0.001)
LDH (U/L)	0.395 (<0.001)
HDL (mmol/L)	−0.344 (<0.001)
Triglycerides (mmol/L)	0.178 (0.08)
CRP (mg/L)	0.330 (<0.001)
hsCRP (mg/L)	0.414 (<0.001)
Proteinuria (mg/L)	0.434 (0.01)
Creatinine clearance (mL/min)	−0.496 (<0.001)

Abbreviations: eGFR CKD-EPI, estimated glomerular filtration rate chronic kidney disease epidemiology collaboration equation; LDH, lactate dehydrogenase; HDL, high-density lipoprotein; CRP, C-reactive protein; hsCRP, high-sensitivity C-reactive protein.

**Table 5:** Predicting the likelihood of higher calprotectin values (>1.4); bivariate logistic regression.

Bivariate Logistic Regression	ß	*p*	OR (95% CI)
Respiratory insufficiency	2.39	0.03	11 (1.30–93.3)
Acute kidney failure	1.09	0.03	3 (1.10–8.17)
Time since COVID-19 (<6 months)			
6–12 months	−1.32	0.01	0.27 (0.096–0.74)
>12 months	-	>0.99	-
Calcineurin inhibitor (none)			
Ciclosporin	1.09	0.12	2.96 (0.75–11.67)
Tacrolimus	0.73	0.26	2.06 (0.58–7.28)
Adjacent (none)			
Micophenolate–mofetil	−0.28	0.67	0.76 (0.20–52.80)
Azathioprine	−19.5	0.99	-
mTOR inhibitor (none)			
Everolimus	−0.97	0.12	0.38 (0.11–1.28)
Sirolimus	−0.57	0.65	0.57 (0.05–6.52)
Steroids (none)			
5 mg/4 mg	−1.13	0.37	0.32 (0.03–3.71)
10 mg	0.69	0.68	2.0 (0.08–51.6)
15 mg	18.5	0.99	-
Calcium channel blocker	1.09	0.03	3 (1.13–7.96)
Moxonidine	1.16	0.007	3.2 (1.38–7.4)
Urapidil	1.06	0.03	2.9 (1.12–7.52)
Valganciclovir	1.26	0.05	3.51 (0.99–12.4)
Leukocytes (×10^9^)	0.26	0.009	1.29 (1.07–1.59)
Neutrophils (%)	0.07	0.003	1.07 (1.02–1.12)
Lymphocytes (%)	−0.07	0.005	0.93 (0.88–0.98)
Urea (mmol/L)	0.10	0.009	1.11 (1.03–1.20)
Creatinine (µmol/L)	0.007	0.02	1.01 (1.001–1.01)
eGFR CKD-EPI (mL/min/1.73 m^2^)	−0.03	0.006	0.97 (0.95–0.99)
LDH (U/L)	0.01	0.009	1.01 (1.003–1.02)
HDL (mmol/L)	−1.22	0.02	0.29 (0.10–0.83)
Phosphorus (mmol/L)	1.35	0.05	3.85 (0.98–15.2)
Creatinine clearance (mL/min)	−0.03	0.04	0.96 (0.94–0.99)
Ratio neutrophils/lymphocytes	0.41	0.02	1.50 (1.07–2.09)

Abbreviations: CI, confidence interval, OR, odds ratio; mTOR, mammalian target of rapamycin; eGFR CKD-EPI, estimated glomerular filtration rate chronic kidney disease epidemiology collaboration equation; LDH, lactate dehydrogenase; HDL, high-density lipoprotein.

**Table 6:** Predicting the likelihood of higher calprotectin values (>1.4); multivariate logistic regression–stepwise method.

Multivariate Logistic Regression	ß	*p*	OR (95% CI)
Leukocytes (×10^9^)	0.48	0.03	1.62 (1.03–2.6)
eGFR CKD-EPI (mL/min/1.73 m^2^)	−0.05	0.04	0.95 (0.90–0.98)
Constant	−1.01	0.04	

Abbreviations: ß—regression coefficient; OR, odds ratio; CI, confidence interval; eGFR CKD-EPI, estimated glomerular filtration rate chronic kidney disease epidemiology collaboration equation.

## Data Availability

Data are available upon request from the corresponding author’s e-mail.

## Supplementary Materials

The following supporting information can be downloaded at: https://www.mdpi.com/article/10.3390/v15081776/s1, title; Supplementary Table S1. Differences in long COVID symptoms according to serum calprotectin level; Supplementary Table S2. Differences in laboratory data according to serum calprotectin level, Supplementary Table S3. Predicting the likelihood of higher calprotectin values (>1.4); bivariate logistic regression.
