# Supplementary material for: Associations of Serum Calprotectin, Arterial Stiffness and Long COVID Symptoms in Dalmatian Kidney Transplant Recipients
Source: Viruses. 2023 Aug 21;15(8):1776. doi: 10.3390/v15081776 (PMC10458603; doi:10.3390/v15081776)
Supplement: Supplementary file 1 [file viruses-15-01776-s001.zip › viruses-2533001-supplementary.pdf]

Supplementary Table S1. Differences in long COVID symptoms according to serum calprotectin level

	Number (%) of patients			P*
	Low calprotectin ≤ 1.4 (n = 56)	High calprotectin > 1.4 (n = 42)	Total (n = 98)	
Mobility				
No	29 (51.8)	25 (61)	54 (55.7)	0.67†
Slight	10 (17.9)	6 (14.6)	16 (16.5)	
Moderate	9 (16.1)	7 (17.1)	16 (16.5)	
Severe and unable	8 (14.3)	3 (7.3)	11 (11.3)	
Self-care				
No	51 (91.1)	40 (97.6)	91 (93.8)	0.43†
Slight	3 (5.4)	0 (0)	3 (3.1)	
Moderate	1 (1.8)	1 (2.4)	2 (2.1)	
Severe and unable	1 (1.8)	0 (0)	1 (1)	
Usual activities				
No	34 (60.7)	29 (70.7)	63 (64.9)	0.86†
Slight	7 (12.5)	5 (12.2)	12 (12.4)	
Moderate	11 (19.6)	5 (12.2)	16 (16.5)	
Severe and unable	4 (7.2)	2 (4.9)	6 (6.2)	
Pain/discomfort				
No	25 (44.6)	21 (51.2)	46 (47.4)	0.51†
Slight	10 (17.9)	9 (21.9)	19 (19.5)	
Moderate	13 (23.2)	9 (22)	22 (22.7)	
Severe and extreme	8 (14.3)	2 (4.9)	10 (10.3)	
Anxiety/depression				
Not	38 (67.9)	33 (80.5)	71 (73.2)	0.17†

Slightly	12 (21.4)	6 (14.6)	18 (18.6)
Moderately	6 (10.7)	1 (2.4)	7 (7.2)
Severely and extremely	0 (0)	1 (2.4)	1 (1)

	Median (IQR)			P*
	Low calprotectin ≤ 1.4 (n = 56)	High calprotectin > 1.4 (n = 42)	Total (n = 98)	
Mobility	1 (1 - 3)	1 (1 – 2.5)	1 (1 - 3)	0.33
Self-care	1 (1 - 1)	1 (1 - 1)	1 (1 - 1)	0.20
Usual activities	1 (1 - 3)	1 (1 - 2)	1 (1 - 2)	0.27
Pain/discomfort	2 (1 - 3)	1 (1 - 3)	2 (1 - 3)	0.22
Anxiety/depression	1 (1 - 2)	1 (1 - 1)	1 (1 - 2)	0.16
EQ-VAS %	79 (52.5 - 80)	80 (62.5 - 90)	80 (60 - 90)	0.12
mMRC dyspnoea scale	1 (0 - 1)	0 (0 - 1)	0 (0 - 1)	0.51

*χ^2 test; †Fisherov egzaktni test *Mann Whitney U test

Abbreviations: EQ-5D-5L, Europian quality of life group 5-dimension 5-level health questionnaire; IQR, interquartile range; EQ-5D-5L, Europian quality of life group 5-dimension 5-level health questionnaire; EQ-VAS, Europian quality of life visual analogue scale; mMRC, modified Medical Research Council

Supplementary Table S2. Differences in laboratory data according to serum calprotectin level

	Median (IQR)			P*
	Low calprotectin ≤ 1.4 (n = 56)	High calprotectin > 1.4 (n = 42)	Total (n = 98)	
Leukocytes (10^9/L)	6.7 (5.73 – 7.5)	8 (5.78 – 10.5)	6.95 (5.78 – 8.73)	**0.02**
Erythrocytes (10^{12}/L)	4.8 (4.26 – 5.32)	4.58 (4.06 – 5.39)	4.7 (4.21 – 5.35)	0.40
Hemoglobin (g/L)	134.5 (123.25 – 147.75)	133.5 (119 - 151)	134 (123 - 150)	0.68
Hematocrit (L/L)	0.43 (0.39 – 0.46)	0.41 (0.36 – 0.47)	0.42 (0.38 – 0.47)	0.36
MCV (fL)	87.85 (84.75 – 91.35)	89.4 (84.8 – 92.93)	88.45 (85.05 – 92.43)	0.49

Platelets (10^9/L)	199 (157.5 – 248.5)	220 (182.5 - 248)	207 (171.5 - 248)	0.13
Neutrophils (%)	56.6 (49.7 – 65.9)	64.05 (57.05 – 69.68)	60.7 (53.75 – 67.25)	**0.004**
Lymphocytes (%)	30.2 (21.9 - 36)	24.6 (17.3 – 31.03)	27.9 (20.75 – 34.05)	**0.005**
Monocytes (%)	9.4 (7.9 – 10.8)	9.05 (7.5 – 11.03)	9.3 (7.6 – 10.85)	0.35
Eosinophils (%)	1.4 (0.9 – 2.2)	1.3 (0.58 – 2.25)	1.3 (0.75 – 2.2)	0.41
Basophils (%)	0.5 (0.3 – 0.7)	0.4 (0.3 – 0.6)	0.4 (0.3 – 0.6)	0.30
Glucose (mmol/L)	5 (4.5 – 5.5)	5.3 (4.6 - 7)	5.2 (4.55 – 5.95)	0.11
Urea (mmol/L)	7.55 (5.93 – 10.8)	10.6 (8.08 – 17.48)	9.1 (6.43 – 12.35)	**0.003**
Creatinine (µmol/L)	118.5 (94.5 - 147)	144 (112.75 – 210.75)	123.5 (100 - 169)	**0.003**
eGFR CKD-EPI (mL/min/1.73 m^2)	51.6 (39.63 – 66.15)	41.75 (25.33 – 56.65)	44.3 (34.58 – 63.6)	**0.007**
Urates (µmol/L)	355.5 (305 – 410.75)	376 (341.5 - 418)	363 (324 – 414.5)	0.14
AST (U/L)	21 (18 - 28)	19 (15 – 25.25)	21 (17 - 26)	0.07
ALT (U/L)	19 (16 - 29)	21.5 (14.75 - 29)	21 (16 - 29)	0.95
GGT (U/L)	22 (16 - 33)	25.5 (16 - 42)	23 (16 - 38)	0.38
LDH (U/L)	187.5 (170.75 – 213.75)	210 (185.5 – 250.5)	198 (174 - 229)	**0.009**
ALP (U/L)	78 (60 - 94)	80 (60.75 - 106)	79 (60.5 – 98.5)	0.53
Iron (µmol/L)	15 (13 - 18)	14 (10 - 16)	14 (12 - 18)	0.17
UIBC (µmol/L)	36 (31 - 41)	34 (28.5 - 42)	35 (30 – 41.75)	0.68
TIBC (µmol/L)	50 (47 - 57)	49 (42 - 57)	50 (44.25 - 57)	0.64
Tsat (%)	30 (24 - 38)	28 (22.5 - 35)	29.5 (24 - 35)	0.31
Ferritin (ng/mL)	121 (63 - 240)	117 (51 - 262)	121 (59 - 253)	0.68
Cholesterol (mmol/L)	4.8 (4.3 – 5.6)	4.9 (4.1 – 5.95)	4.85 (4.3 – 5.68)	0.92
HDL (mmol/L)	1.55 (1.3 – 1.8)	1.3 (1 – 1.65)	1.5 (1.2 – 1.7)	**0.01**
LDL (mmol/L)	2.7 (2.05 – 3.23)	2.6 (1.85 – 3.35)	2.7 (1.9 – 3.3)	0.89
Triglycerides (mmol/L)	1.6 (1.1 - 2)	1.7 (1.35 – 2.95)	1.6 (1.13 – 2.2)	0.16

Sodium (mmol/L)	141 (139 - 142)	141 (139 - 142)	141 (139 - 142)	0.82
Potassium (mmol/L)	4.2 (3.8 – 4.5)	4.2 (3.98 – 4.73)	4.2 (3.9 – 4.6)	0.26
Chlorine (mmol/L)	102 (100 - 105)	102 (100.75 – 104.25)	102 (100.5 - 105)	0.67
Calcium (mmol/L)	2.37 (2.27 – 2.48)	2.33 (2.23 – 2.43)	2.37 (2.25 – 2.45)	0.20
Phosphorus (mmol/L)	0.99 (0.82 – 1.15)	1.11 (0.91 – 1.32)	1.04 (0.87 – 1.21)	**0.04**
D-dimers (mg/L)	0.56 (0.36 – 1.18)	0.77 (0.46 – 1.63)	0.62 (0.37 – 1.37)	0.27
Calprotectin (µg/L)	1.2 (0.8 – 1.5)	3.85 (2.58 – 5.43)	1.7 (1.1 – 3.45)	**<0.001**
CRP (mg/L)	1.7 (1 – 4.85)	3.1 (1.7 – 8.5)	2.5 (1.2 – 5.95)	**0.02**
hsCRP (mg/L)	1.65 (0.73 – 4.53)	3.43 (1.58 – 8.9)	1.98 (0.93 – 5.79)	**0.005**
Total proteins (g/L)	65 (62 - 68)	64.5 (62 - 70)	65 (62 – 69.25)	0.85
Albumins (g/L)	43.2 (41.33 – 45.13)	44.2 (42.98 – 46.03)	43.95 (41.58 – 45.65)	0.17
ACR (mg/mmol)	4.71 (1.52 – 14.02)	13.42 (2.25 – 60.5)	6.34 (1.6 – 44.51)	0.10
PCR (mg/mmol)	16.45 (9.01 – 31.37)	36.56 (9.36 – 99.38)	18.41 (9.01 – 61.93)	0.09
Albuminuria (mg/L)	41 (19.25 – 688.25)	129 (43 - 953)	58 (28 - 864)	0.13
Proteinuria (mg/L)	187 (140 – 693.5)	436 (203 - 1210)	242 (155.5 - 1080)	0.07
Creatinine clearance (mL/min)	58.4 (50.6 – 78.35)	49.07 (25.22 – 60.41)	57.63 (45 – 72.73)	**0.04**
Spot urine – Erythrocytes	0.5 (0 - 2)	1 (0 - 2)	1 (0 - 2)	0.83
Spot urine – Leukocytes	1 (0 - 2)	1 (0 - 1)	1 (0 - 2)	0.22

*Mann Whitney U test

Abbreviations: IQR, interquartile range; MCV, mean corpuscular volume; eGFR CKD-EPI, estimated glomerular filtration rate chronic kidney disease epidemiology collaboration equation; AST, aspartate aminotransferase; ALT, alanine transaminase; GGT, gamma-glutamyl transferase; LDH, lactate dehydrogenase; ALP, alkaline phosphatase; UIBC, unsaturated iron-binding capacity; TIBC, total iron-binding capacity; Tsat, transferrin

saturation; HDL, high-density lipoprotein; LDL, low-density lipoprotein; CRP, C-reactive protein; hsCRP, high-sensitivity C-reactive protein; ACR, urine albumin to creatine ratio; PCR, urine protein to creatinine ratio

Supplementary Table S3. Predicting the likelihood of higher calprotectin values (> 1.4); bivariate logistic regression

**Bivariate logistic regression**	ß	P	OR (95% CI)
Sex (F)	-0.37	0.39	0.69 (0.29 – 1.61)
Respiratory insufficiency	2.39	**0.03**	11 (1.30 – 93.3)
Acute kidney failure	1.09	**0.03**	3 (1.10 – 8.17)
Diabetes in acute COVID-19	1.15	0.06	3.15 (0.96 – 10.27)
Dyspnoea	20.7	0.99	-
Basal proteinuria (mg/L)	0.002	0.07	1.002 (0.99 – 1.004)
Basal albuminuria (mg/L)	0.003	0.09	1.003 (0.99 – 1.01)
Time since COVID-19 (< 6 months)			
6 – 12 months	-1.32	**0.01**	0.27 (0.096 – 0.74)
> 12 months	-	>0.99	-
Calcineurin inhibitor (none)			
Cyclosporine	1,09	0,12	2,96 (0,75 – 11,67)
Tacrolimus	0,73	0,26	2,06 (0,58 – 7,28)
Adjacent (none)			
Micophenolate-mofetil	-0,28	0,67	0,76 (0,20 – 52,80)
Azathiprin	-19,5	0,99	-
mTOR inhibitor (none)			
Everolimus	-0,97	0,12	0,38 (0,11 – 1,28)
Sirolimus	-0,57	0,65	0,57 (0,05 – 6,52)
Steroids (none)			
5mg/4mg	-1,13	0,37	0,32 (0,03 – 3,71)

10 mg	0,69	0,68	2,0 (0,08 – 51,6)
15 mg	18,5	0,99	-
Beta-blockers	0.86	0.09	2.36 (0.88 – 6.35)
CCB	1.09	**0.03**	3 (1.13 – 7.96)
Diuretics	0.29	0.50	1.33 (0.58 – 3.09)
Moxonidine	1.16	**0.007**	3.2 (1.38 – 7.4)
Urapidil	1.06	**0.03**	2.9 (1.12 – 7.52)
Minoxidil	20.5	0.99	-
Valganciclovir	1.26	0.05	3.51 (0.99 – 12.4)
Leukocytes (x10^9/L)	0.26	**0.009**	1.29 (1.07 – 1.59)
Neutrophils (%)	0.07	**0.003**	1.07 (1.02 – 1.12)
Lymphocytes (%)	-0.07	**0.005**	0.93 (0.88 – 0.98)
Basophils (%)	-1.27	0.13	0.28 (0.05 – 1.48)
Glucose (mmol/L)	0.31	0.06	1.36 (0.99 – 1.86)
Urea (mmol/L)	0.10	**0.009**	1.11 (1.03 – 1.20)
Creatinine (µmol/L)	0.007	**0.02**	1.01 (1.001 – 1.01)
eGFR CKD-EPI (mL/min/1.73 m^2)	-0.03	**0.006**	0.97 (0.95 – 0.99)
LDH (U/L)	0.01	**0.009**	1.01 (1.003 – 1.02)
HDL (mmol/L)	-1.22	**0.02**	0.29 (0.10 – 0.83)
Phosphorus (mmol/L)	1.35	0.05	3.85 (0.98 – 15.2)
CRP (mg/L)	0.06	0.06	1.06 (0.99 – 1.22)
hsCRP (mg/L)	0.05	0.06	1.05 (0.99 – 1.09)
Creatinine clearance (mL/min)	-0.03	**0.04**	0.96 (0.94 – 0.99)
Albuminuria (mg/L)	0.001	0.27	1.001 (0.99 – 1.002)
Proteinuria (mg/L)	0.001	0.19	1.001 (0.99 – 1.002)
Ratio neutrophils / lymphocytes	0.41	0.02	1.50 (1.07 – 2.09)

Ratio CRP/ albumins	2.14	0.07	8.53 (0.81 – 89.4)

Abbreviations: OR, odd ratio; CI, confidenece interval; F, female; mTOR, mammalian target of rapamycin; CCB, calcium channel blockers; eGFR CKD-EPI, estimated glomerulal filtration rate chronic kidney disease epidemiology collaboration equation; LDH, lactate dehydrogenase; HDL, high-density lipoprotein; CRP, C-reactive protein; hsCRP, high-sensitivity C-reactive protein
